# Oscillometric measurement of blood pressure: a simplified explanation. A technical note on behalf of the British and Irish Hypertension Society

**DOI:** 10.1038/s41371-019-0196-9

**Published:** 2019-03-29

**Authors:** Philip S. Lewis, on behalf of the British and Irish Hypertension Society’s Blood Pressure Measurement Working Party, N Chapman, P Chowienczyk, C Clark, E Denver, P Lacy, U Martin, R McManus, A Neary, J. Sheppard

**Affiliations:** Stockport NHS Foundation Trust, Stockport and University of Manchester, Manchester, UK

**Keywords:** Health care, Physiology

## Introduction

It is often assumed that automatic and manual blood pressure (BP) measurements are the same. This can lead to significant errors in BP estimation resulting from differences in technology and methodology. This article explains in simplified terms the way in which most automated oscillometric BP machines measure BP and how it differs from manual BP measurement. The aim is to explain some of the limitations of the oscillometric method and to encourage use of correctly validated machines.

## Auscultatory measurement of blood pressure

The time-honoured, traditional measurement of BP uses an inflatable cuff encircling a limb combined with a stethoscope to detect sounds made by the flow of blood as it comes under the cuff (the Riva-Rocci—Korotkoff auscultatory method) [1, 2]. The cuff is inflated to a point where the pressure it exerts on the underlying arm is high enough to stop blood flowing underneath so that no blood flow sounds can be heard. As the cuff pressure is deflated the pressure transmitted from the cuff to the walls of the underlying arteries reduces to the point at which blood flow resumes and sounds begin to be heard. These sounds vary in intensity and usually stop at the point of the lowest pressure within the arteries before the next pulse arrives. The initial sound approximates to the peak (systolic) and the final sound to the relaxation (diastolic) pressure in the artery [2]. Listening to these sounds and interpreting them requires training and can be difficult especially if hearing is impaired or there is significant environmental noise.

## Oscillometric measurement

**Fig. 1:**
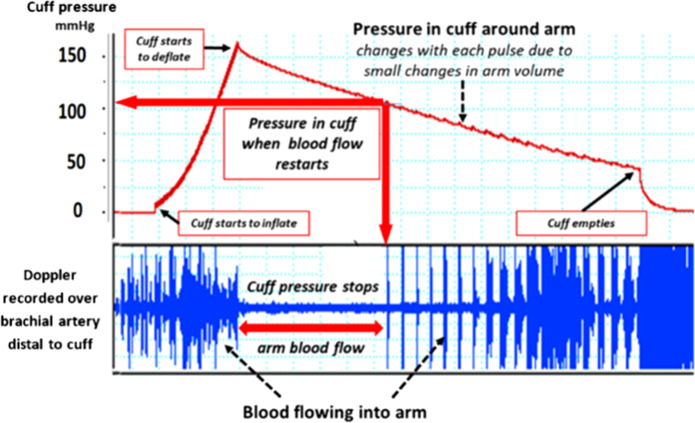
The relationship between the pressure in an automated machine cuff and the flow of blood into the underlying arm

Knowing how cuff-only oscillometric readings are estimated helps in understanding the limitations of this method. With every arterial pulse wave there is a small rise and fall in the volume of the limb, which in turn causes an increase and then a decrease in the pressure within the encircling cuff, which can be detected using a solid-state transducer. When the cuff encircling a limb is inflated with an electronic pump (or sometimes manually), the rising pressure in the cuff eventually stops arterial blood flowing into the underlying limb and pulsation ceases. This is detected by the machine which continues to inflate the cuff for a second or two more to ensure that the limb flow has stopped completely. At this point, inflation stops, and a valve opens allowing the pressure in the cuff to reduce slowly (Fig. 1). The pressure within the cuff is monitored carefully by the machine. At first it only detects the pulseless reduction in pressure. As the pressure in the cuff falls to below the pressure of the peak of the arterial pulse, the machine begins to detect a small pressure wave which reflects the difference between the pressure in the cuff and that in the artery. With further cuff deflation these pressure differences become greater until the cuff begins to fall away from the limb and less of the volume pulsation is detected. The machine therefore records within it a series of pulse waves, which are initially flat, then very slight, then increase to a peak and then diminish until they are hardly detected.

**Fig. 2:**
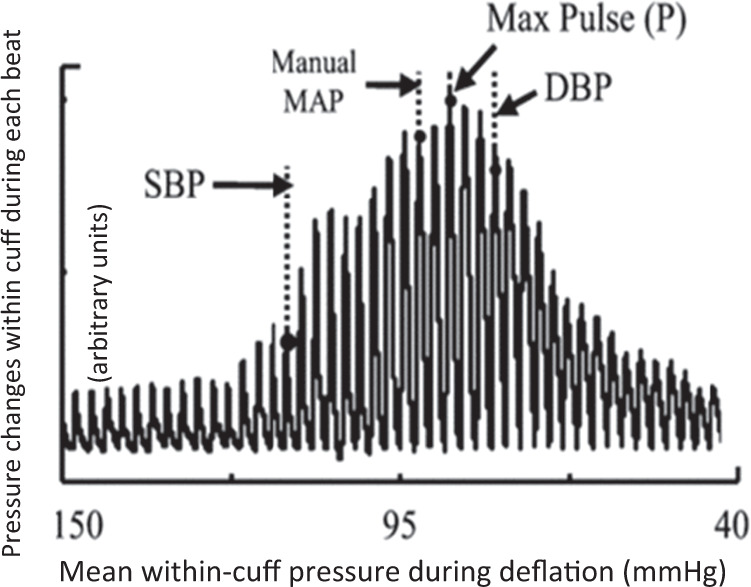
Cuff pressure changes used to calculate blood pressure [5]

Some machines read pressure and volume changes during cuff inflation until the arm stops pulsating after which the cuff rapidly deflates. Oscillometric machines usually use the maximum volume change as an indication of the average of the systolic and diastolic BP within the artery. By combining this average with the rate of change of the pressure wave, the machines then use a variety of algorithms to estimate the systolic and diastolic BP. These algorithms vary from machine to machine resulting in slightly different interpretations of the pressures (Fig. 2).

Pulse detection by oscillometric machines depends on the amount of change in the volume of the arm with each pulse (small pulses are more difficult to detect) and on the regularity and rate of those pulses. With regular pulses and a relatively smoothly changing arm volume it is much easier for the microprocessor to estimate the systolic and diastolic BP. However, if the pulses are irregular or there are movements in the arm under the cuff, pressure changes in the cuff will not rise and fall smoothly leading to difficulty in making the pressure calculations. This can arise with rhythm problems such as atrial fibrillation and when there are frequent extrasystoles which may have a smaller volume than regular beats. Very slow heart rates (<50 bpm) may lead to too few beats being detected during cuff deflation to permit an accurate estimate of BP.

The use of reliable solid-state transducers and microprocessor technology allows these pressure changes in the cuff to be detected and analysed permitting easy access to a fair and reproducible estimation of arterial BP economically and without the need for skilled interpretation.

## Differences between auscultatory and oscillometric blood pressure readings

Automated oscillometric machines differ with respect to their algorithms, transducers, inflation and deflation rates, cuff sizes and materials, all of which may affect the estimation of BP. These may result in significant differences in estimations of systolic and diastolic BP compared with auscultatory readings in the same patient. Some machines may be accurate in one subject group but not necessarily in others e.g. in obese or pregnant subjects. Therefore, it is essential that oscillometric machines are validated against auscultatory readings in specified groups of subjects by experienced observers, approval being given to those machines which are deemed sufficiently accurate. The British and Irish Hypertension Society [3] and other organisations assess and publish lists of successfully validated oscillometric machines. A variety of validation protocols have been used but these are being superseded by a new Universal Standard [4].

Despite these considerations, the development of automated oscillometric BP machines has facilitated access to BP measurement without the need for expert training. BP screening and identification and management of hypertension is now more widely available than ever before.

## Conclusions

Oscillometric BP machines have simplified the measurement of BP and removed the need for expert training. Nevertheless, a basic understanding of the technology of oscillometric reading explains why there are limitations to their accuracy and prompts the need to use machines properly validated for specific population groups and subject characteristics.
