# Correction: Oscillometric measurement of blood pressure: a simplified explanation. A technical note on behalf of the British and Irish Hypertension Society

**DOI:** 10.1038/s41371-020-0316-6

**Published:** 2020-02-26

**Authors:** Philip S. Lewis, on behalf of the British and Irish Hypertension Society’s Blood Pressure Measurement Working Party, N. Chapman, P. Chowienczyk, C. Clark, E. Denver, P. Lacy, U. Martin, R. McManus, A. Neary, J. Sheppard

**Affiliations:** Stockport NHS Foundation Trust, Stockport and University of Manchester, Manchester, UK

**Keywords:** Health care, Physiology

**Correction to: Journal of Human Hypertension**

10.1038/s41371-019-0196-9

This Article was originally published under Nature Research’s License to Publish, but has now been made available under a [CC BY 4.0] license. The PDF and HTML versions of the Article have been modified accordingly.

